# Strangulated Hernia Can Be a Risk Factor of Seroma following Laparoscopic Transabdominal Preperitoneal Repair

**DOI:** 10.1155/2018/6528075

**Published:** 2018-08-26

**Authors:** Ryu Matsumoto, Yoshio Nagahisa, Kazuki Hashida, Mitsuru Yokota, Michio Okabe, Kazuyuki Kawamoto

**Affiliations:** Department of Surgery, Kurashiki Central Hospital 1-1-1, Miwa, Kurashiki, Okayama 710-8602, Japan

## Abstract

**Purposes:**

Seroma is a postoperative complication following laparoscopic transabdominal preperitoneal repair (TAPP) for inguinal hernioplasty. Seroma naturally resolves in most cases, but it can lead to an increased amount of visits to the outpatient clinic and can result in anxiety of the patient. Local inflammation of the inguinal area is etiology of seroma formation. Strangulated hernia involves severe inguinal pain and can lead to severe inflammation and subsequent seroma. There have been no reports demonstrating the links of seroma and strangulated hernia. This study aimed to retrospectively evaluate the risk of seroma after TAPP and to identify the association between strangulated hernia and seroma.

**Methods:**

We treated 300 inguinal hernias by TAPP between 2013 and 2016 at Kurashiki Central Hospital. We used the Chi-square test. Factors significant in each association were further examined using multiple subsequent logistic regressions.

**Results:**

A total of 222 hernias were eligible for analysis. The incidence of seroma was 11% (n=25). There were nine cases of strangulated hernias, and three (33%) resulted in seroma. The ratio of strangulated hernia of seroma group is significantly high (p<0.03). Multiple subsequent logistic regressions showed that strangulated hernia was associated with a significantly increased risk for seroma formation (p = 0.023; OR 6.564; 95% CI 1.29-33.3).

**Conclusion:**

This study shows that strangulated hernia can be a risk factor in seroma formation. This risk should be incorporated into a management plan of TAPP for strangulated hernia, with careful consideration of patients' concerns.

## 1. Introduction

Seroma is a postoperative complication following laparoscopic transabdominal preperitoneal repair (TAPP) for inguinal/ femoral hernioplasty. Seroma naturally resolves in most cases, but it can lead to an increased amount of visits to the outpatient clinic and can result in anxiety of the patients because they misunderstand seroma as recurrence of hernia. A remaining fluid hernia sac during the operation is a major risk of seroma. Moreover, local inflammation of the inguinal area resulting from dissection of the preperitoneal layer and the use of prosthetic materials to cover the myopectineal orifice have been reported as etiologies of seroma formation. However, the precise etiology of seroma needs to be clarified. Strangulated hernia is a common disease, which is often observed in surgery and the emergency room, and involves severe inguinal pain. Strangulated hernia can lead to severe inflammation and subsequent seroma. Consensus on the surgical approach for strangulated hernia is yet to be reached.

This study aimed to retrospectively evaluate the risk of seroma after TAPP and to identify the association between strangulated hernia and seroma. To the best of our knowledge, this is the first report to demonstrate that strangulated hernia can be a risk factor of seroma.

## 2. Materials and Methods

This retrospective study was conducted at one institution. We obtained written informed consent for the inclusion of data from all of the participating patients.

**Figure 1:**
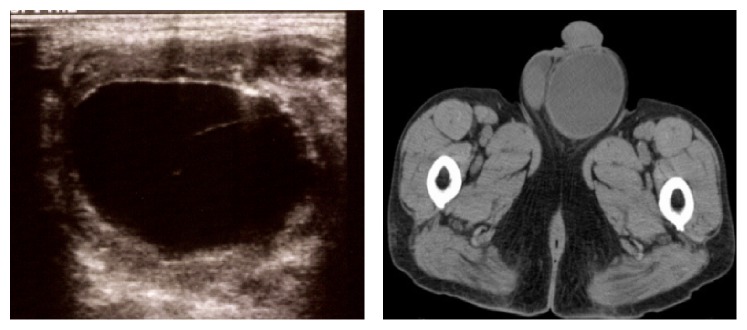
Seroma was diagnosed by ultrasonography (left) or computed tomography (right).

We perform approximately 12,000 elective surgeries and 600 emergent surgeries in our hospital per year. We treated 300 adult inguinal/ femoral hernias by TAPP between 2013 and 2016 at Kurashiki Central Hospital. We excluded patients' data if there was no information on the size of the hernia, which is correlated with the risk of seroma [1]. We also excluded patients with combined surgeries because we could not precisely evaluate the postoperative pain and complications after multiple surgeries. We examined all patients visually for signs of inguinal swelling, and by palpation in the outpatient clinic on postoperative day 7, and further investigation by ultrasonography or computed tomography (CT) was undertaken in patients with findings suggestive of seroma (Figure 1).

The diagnosis of strangulated hernia was based on physical examination such as painful palpable bulge and/or nonreducible mass of the inguinal region. We performed the emergent surgery for the nonreducible hernia, but for the reducible strangulated hernias we performed the early elective surgery (within 48 hours from the diagnosis) after the preoperative evaluation. Laparoscopy allowed for identification of hernia sac contents (small intestine or omentum). Strangulated hernias were reduced using a combination of manual and laparoscopic manipulation. If the resection of small intestine or omentum was required, we firstly performed inguinal repair with mesh. The hernias over 3cm in diameter were defined as large hernias and the hernias less than 3cm were defined as small hernias. The nurse at each surgical unit evaluated the postoperative pain score with the visual analogue scale (VAS) 0 and 6 hours after, and on the next day after the operation. The largest score was defined as VAS MAX. We allocated the cases to two groups (VAS MAX<0 or VAS MAX>1).

We allocated the patients into the seroma and no seroma group. The patient's demographics included gender, age, and body mass index (BMI). Hernia data was collected about position of the hernia, type of hernia, hernia anatomy, strangulated hernia, and size of hernia. Operation time, airway device, intraoperative complication, mesh type, VAS MAX, and analgesic use were examined as intraoperative and postoperative data.

### 2.1. Surgical Procedures

Preoperative antibiotic administration was performed to only the strangulated cases. All surgeries were undertaken through three ports using a rigid endoscope (30°) under general anesthesia. A urinary catheter was used only when required. Abdominal CO_2_ pressure was established as 10 mmHg during the procedure. We dissected the preperitoneal layer using the Sandwich approach, because it is feasible for patients with prominent adhesion and is able to be safely performed by young surgeons [2]. We dissected all the hernia sac and did not retract the transversalis fascia. We used two types of mesh: L size 3DMax mesh (Bard, Cranston, RI) and M size Parietex anatomical mesh (Covidien, Mansfield, MA). In strangulated hernias, the same mesh was also used after evaluation as a clean operation. All mesh was fixed with AbsorbaTack (5-mm fixation device; Covidien) at least on the Cooper's ligament and transverse abdominal muscle. After fixation with mesh, we added dissection if needed to reduce the redundant organ incision and the operation time. The peritoneal flap was closed with 3-0 Vicryl in all cases. All surgeries were performed by surgical residents with 3–5 years of experience, and who were supervised by an experienced doctor who also operated as a scopist.

### 2.2. Statistical Analyses

All statistical analyses were performed with Statistical Package for Social Scientists software version 21.0. Associations between position of the hernia, type of hernia, hernia anatomy, strangulated hernia, size of hernia, airway device, mesh type, vas max, and analgesic use and seroma were calculated by Chi-square test. P<0.05 was considered as statistically significant. Factors significant in each association were further examined using multiple subsequent logistic regressions.

## 3. Results

**Figure 2:**
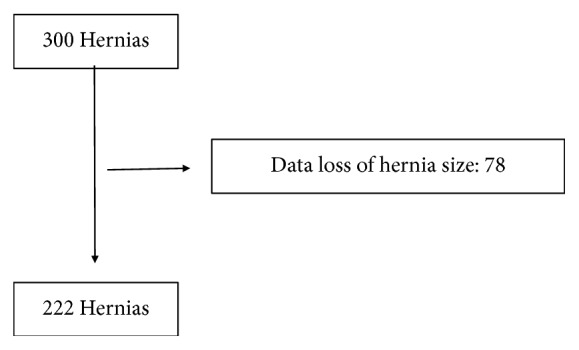
The number of eligible hernias following the exclusion criteria.

After the exclusion, 222 hernias were eligible for analysis (Figure 2).

Table 1 showed the postoperative complications after TAPP.

The incidence of seroma was 11% (n=25) and the recurrent hernia was one (0.4%). One patient showed the peritonitis. The cause of peritonitis was unknown, but it improved with antibiotics. Four patients presented the port site infection, but they improved naturally without antibiotics. Patient clinical characteristics were similar in seroma group and no seroma group (Table 2).

Table 3 showed the hernia data of the two groups.

There were no significant difference with the position of the hernia, type of hernia, and hernia anatomy. There were nine cases of strangulated hernias. In all cases, there were intestine or omentum in the hernia sac. Two patients required the partial resection of the intestine because of bowel ischemia. Three strangulated hernias (33%) resulted in seromas. All cases were indirect hernias. The ratio of strangulated hernia in seroma group was significantly greater than in no seroma group (p<0.03). There was also a significant difference of size of hernia (p<0.02).

Table 4 showed the intraoperative and postoperative data in two study groups. We did not experience any intraoperative complications. There were no significant difference in airway device, mesh type, and analgesic use in two study groups. VAS MAX was significantly high in no seroma group compared to seroma group, but there was no significant difference in analgesic use in two study groups.

Multiple subsequent logistic regressions showed that strangulated hernia was associated with a significantly increased risk for seroma formation (p = 0.023; OR 6.564; 95% CI 1.29-33.3). The size of hernia was statistically borderline as s risk factor (p = 0.055; OR 3.616; 95% CI 0.97-13.4) (Table 5).

## 4. Discussion

We found that TAPP for strangulated hernia could cause seroma. To the best of our knowledge, this is the first study to show that strangulated hernia can be a risk factor for formation of seroma.

A seroma is an accumulation of local fluid, and the incidence of seroma is 3–11% after TAPP [3, 4].

A seroma does not affect the patient's recovery and is considered as a minor complication. However, seroma mimics the symptoms of recurrent hernia. Moreover, the incidence of seroma is higher than that of recurrent hernia following TAPP [1, 5]. Patients often mistake a seroma as a recurrent hernia, which can lead to increased visits to the outpatient clinic. This is a serious problem.

There have been several reports on formation of seroma [1, 6]. A large hernial defect space remaining during surgery plays an important role in formation of seroma. In our research, hernia size was statistically borderline as a risk factor of seroma formation. Additionally, an inflammatory response by preperitoneal preparation in surgery and from the presence of polypropylene mesh has also been reported to cause seroma [1]. However, the mechanisms of seroma formation are still unclear. There have been no reports that specifically demonstrated an association between strangulated hernia and seroma formation. In the present study, multiple subsequent logistic regressions showed that strangulated hernia was a risk factor of seroma formation.

The overall incidence of emergent inguinal hernia repair in patients undergoing watchful waiting is 2.5% over 10 years [7]. In patients with strangulated hernia, we first attempt to reduce the strangulated hernia, but if we cannot reduce the hernia, emergent surgery or an early elective surgery is necessary. These operations are performed during the occurrence of severe inflammation. Therefore, in addition to local inflammation resulting from hernial strangulation, surgical dissection and mesh implantation may cause a further inflammatory response, which could lead to formation of seroma.

However, the incidence of seroma varies depending on each physician [8], because an objective examination, such as ultrasound and computed tomography, is generally not performed [1]. Furthermore, consensus is yet to be reached about the appropriate timing of diagnosis of seroma. A seroma is considered as a normal postoperative physiological phenomenon. Therefore, the incidence of seroma immediately following surgery is high [6, 9]. Parl et al. suggested that seroma should be diagnosed only if it is symptomatic and persistent beyond 6 weeks [10]. In our series, seroma formation was detected based on an US examination or CT for visible inguinal swelling at postoperative day 7 to ensure correct diagnosis and evaluation of its incidence.

Awareness of strangulated hernias as a risk factor of seroma and its inclusion in a management plan are important. First, consensus has yet to be reached on the best surgical approach of strangulated hernias [5, 11, 12]. Schmedt et al. showed that Lichtenstein repair resulted in a lower incidence of seroma formation than endoscopic procedures [12]. TAPP results in more seroma formation than laparoscopic total extraperitoneal repair [13]. However, these reports did not focus on formation of seroma as the primary goal. In our hospital, we usually perform TAPP for strangulated hernias because of its safety and have experienced no incidence of conversion to open repair or organ injury. Therefore, we consider TAPP as a feasible approach for repair of strangulated hernia. However, an operative approach should not be determined based on the risk of seroma alone. The best approach should be decided with the patient after a clear explanation about the risk and management of seroma. Second, minimization of the potential space of fluid collection is important. The International Endohernia Society has stated that, in indirect hernia repair, complete reduction of the hernia sac may eliminate the occurrence of chronic seroma [1, 5]. Additionally, the incidence of seroma in cases of direct hernia can be significantly reduced when the lax transversals fascia is inverted, which is performed without an increase in postoperative pain, despite being tacked to Cooper's ligament [5, 8]. Suture of the lax transversals fascia to Cooper's ligament instead of tacking is also an alternative option, which can reduce costs [5]. Third, changing the type of mesh may reduce the formation of seroma.

Macrophages, T cells, and mast cells are the main etiologies of an inflammatory response of mesh insertion. Rosch et al. compared the inflammatory response to polypropylene, prolene, and propylene/polyglactin mesh. In the polypropylene mesh group, inflammation continued for longer than 90 days postoperatively, but polypropylene/polyglactin mesh insertion resulted in lower inflammation and proliferation [14]. However, their study was experimental and conducted on mice. Additionally, a reduction in inflammatory tissue response was not directly associated with a reduction in seroma formation, and the incidence of other complications, such as recurrence and chronic pain, was unclear. Therefore, further research on this issue is necessary. Management of seroma does not always require an invasive procedure. If patients complain about inguinal discomfort and pain because of inguinal swelling, percutaneous aspiration of the fluid may be useful, but this involves a risk of infection of the region proximal to the mesh site [5]. In our series, we experienced one case of seroma aspiration, but, in all other patients, this resolved without the need for intervention. We should inform the patients about the possible seroma, especially after the strangulated hernia repair, and advise them to do self-assessment through palpation, which will resolve the insecurity.

There are some limitations of this study. First, this was a retrospective study. Second, the sample size was small and may have caused subsequent allocation bias. However, we perform many elective and emergent surgeries in our hospital and we think this study can be generalized to the hernia patients.

Third, hernia size was well-known as a risk factor of seroma formation, but this study did not show the significant difference in multiple subsequent logistic regressions. Fourth, the patients with strangulated hernia cause ascitic fluid because of local inflammation, which may accumulate in the hernia space and lead to seroma. This study did not analyze the ascites of strangulated hernia. Therefore, we think further study will be necessary.

## 5. Conclusion

Strangulated hernia can be a risk factor in seroma formation. This risk should be incorporated into a management plan of TAPP for strangulated hernia, with careful consideration of patients' concerns.

## Figures and Tables

**Table 1:** Postoperative complications following TAPP.

Complications	N(%)
Seroma	25(11)

Recurrence	1(0.4)

Port site infection	4(1.6)

Subcutaneous bleeding	1(0.4)

Peritonitis	1(0.4)

Intraabdominal bleeding	1(0.4)

Total	30(13)

**Table 2:** Patients' characteristics.

	Seroma group	No seroma group
Gender		

Male (%)	24 (96)	162 (82)

Female (%)	1 (4)	35 (16)

Total	25	197

Age		

Median (range), years	72 (52-89)	73 (21-100)

BMI		

Mean (± SD), kg/m^2^	22.6 (±2.83)	22.8 (±2.84)

BMI: Body Mass Index

**Table 3:** Hernia data.

	Seroma group	No seroma group	*P* value
Position of the hernia			0.079

Right (%)	12 (48)	114 (58)	

Left (%)	13 (52)	83 (42)	

Total	25	197	

Type of hernia			0.87

Primary (%)	19 (76)	16 (8)	

Recurrent (%)	6 (24)	181 (92)	

Total	25	197	

Hernia anatomy			0.67

Indirect (%)	14 (56)	122 (62)	

Direct (%)	9 (36)	50 (25)	

Femoral (%)	1 (4)	15 (7)	

Other (%)	1 (4)	10 (5)	

Total	25	197	

Strangulated hernia (%)	3 (12)	6 (3)	0.036*∗*

Size of hernia			0.022*∗*

small (≦3cm) (%)	21 (84)	188 (95)	

Large (>3cm) (%)	4 (16)	9 (5)	

Total	25	197	

*∗* Statistically significant

**Table 4:** Intraoperative and postoperative data.

	Seroma group	No seroma group	*P* value
Operation time			

Median, minute	94	93	

Airway device			0.267

SGA(%)	10 (40)	101 (51)	

ETT(%)	15 (60)	96 (49)	

Total	25	197	

Intraoperative complication	0	0	

Mesh type			0.94

Anatomical mesh (%)	14 (56)	109 (54)	

3D mesh (%)	11 (44)	88 (45)	

Total	25	197	

Vas Max			0.014*∗*

0 (%)	9 (36)	30 (15)	

>1 (%)	16 (64)	167 (85)	

Total	25	197	

Analgesic use	6 (24)	85 (43)	0.08

*∗* Statistically significant, ETT: Endotracheal tube, SGA: supraglottic airway

VAS: Visual Analogue Scale

**Table 5:** Risk factors (Odds Ratio, 95% confidence interval, and p value) for the seroma formation after laparoscopic transabdominal preperitoneal repair.

	OR	95% CI	*p* value
The size of hernia	3.616	0.97-13.4	0.055

Strangulated hernia	6.564	1.29-33.3	0.023*∗*

VAS MAX	0.424	0.15- 1.168	0.097

*∗* Statistically significant, VAS: Visual Analogue Scale

## Data Availability

The excel data used to support the findings of this study were supplied by Kurashiki Central Hospital under license and so cannot be made freely available. Requests for access to these data should be made to Ryu Mastumoto (rm15367@kchnet.or.jp).
